# Receptive field sizes and neuronal encoding bandwidth are constrained by axonal conduction delays

**DOI:** 10.1371/journal.pcbi.1010871

**Published:** 2023-08-11

**Authors:** Tim C. Hladnik, Jan Grewe

**Affiliations:** 1 Institute for Neurobiology, Eberhardt Karls Universität Tübingen, Tübingen, Germany; 2 Systems Neurobiology, Werner Reichard Center for Integrative Neurobiology, Universität Tübingen, Tübingen, Germany; University of California at Berkeley, UNITED STATES

## Abstract

Studies on population coding implicitly assume that spikes from the presynaptic cells arrive simultaneously at the integrating neuron. In natural neuronal populations, this is usually not the case—neuronal signaling takes time and populations cover a certain space. The spread of spike arrival times depends on population size, cell density and axonal conduction velocity. Here we analyze the consequences of population size and axonal conduction delays on the stimulus encoding performance in the electrosensory system of the electric fish *Apteronotus leptorhynchus*. We experimentally locate p-type electroreceptor afferents along the rostro-caudal body axis and relate locations to neurophysiological response properties. In an information-theoretical approach we analyze the coding performance in homogeneous and heterogeneous populations. As expected, the amount of information increases with population size and, on average, heterogeneous populations encode better than the average same-size homogeneous population, if conduction delays are compensated for. The spread of neuronal conduction delays within a receptive field strongly degrades encoding of high-frequency stimulus components. Receptive field sizes typically found in the electrosensory lateral line lobe of *A. leptorhynchus* appear to be a good compromise between the spread of conduction delays and encoding performance. The limitations imposed by finite axonal conduction velocity are relevant for any converging network as is shown by model populations of LIF neurons. The bandwidth of natural stimuli and the maximum meaningful population sizes are constrained by conduction delays and may thus impact the optimal design of nervous systems.

## Author summary

Reading out the activity of a population of neurons can yield a more complete picture of the stimulus space. Generally, increasing population sizes increases the amount of information. This applies to homogeneous populations of similarly tuned neurons as well as to heterogeneous ones. So far studies on population coding assumed that information reaches the integrating neuron simultaneously. But neurons are neither disembodied nor does neuronal signaling happen instantaneously; populations cover a certain space and axonal conduction takes time. The interplay of encoding bandwidth, receptive field, respective population, size and axonal conduction velocity has not been addressed before. Our results from the electrosensory system and of model simulations show that stimulus dynamics, receptive field sizes, and conduction velocity have to be factored in when we want to understand the design of nervous systems.

## Introduction

Convergence is a basic network motif in nervous systems. Postsynaptic neurons integrate inputs from multiple presynaptic cells. Combining presynaptic inputs can be beneficial by averaging out independent noise and increasing the information carried by the integrated response. This is, for example, observed in the retinae of vertebrates and invertebrates where neighboring photoreceptors are coupled to improve vision [1, 2]. The total information may be limited by correlations in the presynaptic populations [3–6] and has been the topic of a large host of theoretical and experimental work [7]. If the input population is heterogeneous in the sense that different neurons encode different aspects of the stimulus space, the integrated response can provide a more complete representation of the stimulus space. Such a coding scheme is observed in color vision, olfaction or in the downstream processing in the electrosensory system, where the stimulus space is encoded in separate channels [8–10]. However, heterogeneity can also refer to variations in the encoding properties within a given cell type [4, 10–13]. Modeling and experimental approaches have demonstrated that in such cases the population response can carry more information about the stimulus than homogeneous populations of the same size [4, 11, 14–16], support the extraction of additional response features [9, 17] and be more efficient with respect to the energetic costs of neuronal activity [10].

**Fig 1:**
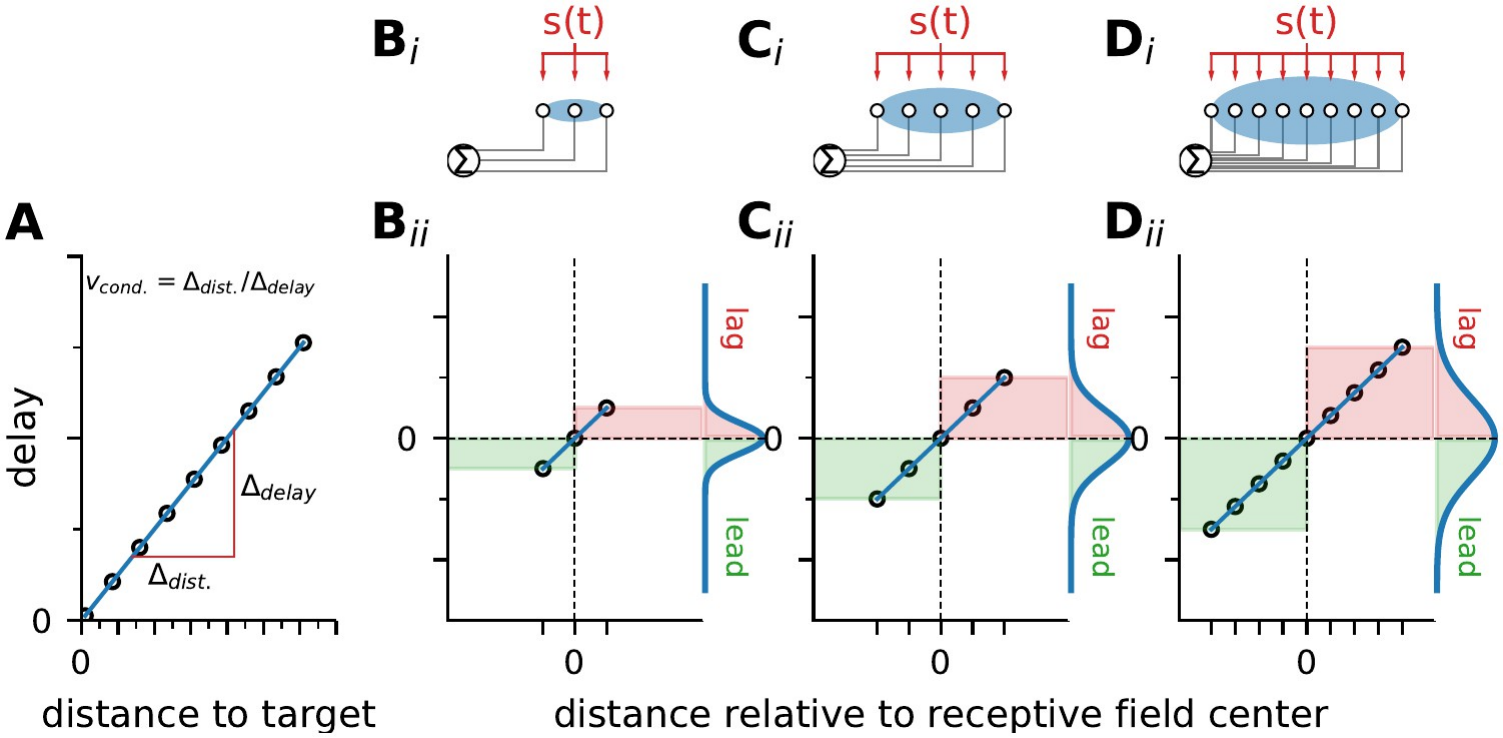
Spread of conduction delays depends on population size. A: Action potential traveling takes time. Depending on the conduction velocity of the nerve fibre (*v*_*cond*._) action potential arrivals at the target will be delayed depending on the travel distance. B_*i*_—D_*i*_: Sketches of the integration of inputs from three differently sized receptive fields (blue ellipses). All neurons of the presynaptic population are driven by the same stimulus (*s*(*t*)) and project to an integrating neuron. The length of the axons connecting the pre- and postsynaptic neurons will vary with the receptor’s location in the receptive field. B_*ii*_—D_*ii*_: Depending on the spatial extent of the receptive field, which is proportional to the size of the presynaptic population, the information from one end of the population will reach the integrating neuron earlier than from the other end. Viewed from the center of the population, the first will lead while the latter will lag behind (green and red areas, respectively). Marginal plots show the probability density of the respective delays among the postsyanptic neuron’s inputs.

Studies considering population coding usually combine presynaptic signals as if they were arriving simultaneously at the integrating neuron. This is not necessarily the case since neuronal signaling is not instantaneous. Rather, information contained in the responses from one end of the receptive field may lead, while the action potentials originating from the other end may lag behind (Fig 1). The spread of conduction delays will depend on the receptive field size and the conduction velocity. Conduction velocity, in turn, depends on axon diameter or myolination and varies across modalities, tissues and species [18, 19]. Fast conduction is an investment that has to pay off and, hence, is mostly considered in the sense of minimizing delays and of mediating behaviorally important tasks such as escape responses [20, 21], when necessary for maximizing encoding bandwidth [19, 22], or where the exact timing of a signal is essential for processing (e.g. in bird sound localization, [23, 24]). The consequences of the spread of conduction delays within populations, has, to our knowledge, not been addressed so far.

Here we investigate the interplay of population coding, heterogeneity, conduction delays, and encoding bandwidth in the active electrosensory system of the weakly electric fish *Apteronotus leptorhynchus*. Electric fish experience their electrical surroundings with two types of electrosensory subsystems, the active (tuberous) and passive (ampullary) system [25–28]. The active system and therein the population of p-type electroreceptor afferents have been extensively studied [29]. P-units are encoders of the fish’s electric field amplitude [30–33] and their response properties are very heterogeneous with respect to their spontaneous as well as their stimulus driven activity [17, 32–34]. About 8000 afferents per side of the fish [35] render the body a large sensory surface of heterogeneous and independent neurons [36, 37]. P-units project to the electrosensory lateral line lobe (ELL) in the hindbrain where they terminate in three adjacent segments of the ELL which differ in their spectral tuning as well as receptive field sizes [6, 28, 38–40]. Assuming a constant conduction velocity, the delay with which spikes from electroreceptors arrive at the brain depends on the receptor’s location on the fish’s body (Fig 1A). Hence, target neurons in the ELL will experience varying spreads of delays depending on their receptive field size.

In this study we first relate P-unit response properties and receptive field location along the rostro-caudal body axis. Then, in an information theoretical approach [41], we test whether the observed heterogeneity is indeed beneficial for the encoding of amplitude modulations and analyze the impact of population size and conduction delays on population coding. Finally, we compare and validate the above results to simulations using leaky integrate-and-fire (LIF) neuron models [42].

## Materials and methods

84 P-units were recorded in 13 subjects of *Apteronotus leptorhynchus* of either sex. Fish were of sizes in the range 10.5 to 22.3 cm, body weights varied in the range 4.1 to 14.6 g, EOD-frequencies varied per individual in the range 611 to 875 Hz. P-type electroreceptor afferents were recorded in the posterior branch of the lateral line nerve. P-units were identified by their typical baseline firing properties and their response to amplitude modulations of the fish’s own field.

### Ethics statement

All experimental procedures complied with German and European regulations and were approved by the animal care committee of the Regierungspräsidium Tübingen (file no. ZP 1/16).

### Surgery

In a surgical intervention the lateral line nerve was exposed by opening a small patch of the skin just above the operculum where the nerve comes close to the surface.

Fish were initially anesthetized with 125 mg/l MS-222 (PharmaQ, Fordingbridge, UK) dissolved in water taken from the housing tank and buffered to pH 7.0 using sodium bicarbonate. When deep anesthesia was reached (gill movements ceased) fish were respirated with a constant flow of water through a mouth tube, containing 100 mg/l MS-222 (pH 7.0) to maintain anesthesia during surgery. The lateral line nerve was exposed dorsal to the operculum. Fish were fixed in the setup with a plastic rod glued to the exposed skull bone. The wounds were locally anesthetized with Lidocainehydrochloride 2% (bela-pharm GmbH, Vechta, Germany) before exposing the nerve. Local anesthesia was renewed every two hours by cutaneous application of Lidocaine to the skin surrounding the surgical wounds.

After surgery, fish were immobilized with 0.05 ml 5 mg/ml tubocurarine (Sigma—Aldrich, Steinheim, Germany) injected into the trunk muscles and were then transferred into the recording tank of the setup filled with water from the fish’s housing tank. Respiration was continued without anesthetic. The animals were submerged into the water so that the exposed nerve was just above the water surface (see S1 Fig). Electroreceptors located on the parts above water surface did not respond to electrical stimulation and were excluded from analysis. Water temperature was kept at 26°C.

### Recording

Action potentials of electroreceptor afferents were recorded intracellularly with sharp borosilicate microelectrodes, pulled to a resistance between 50 and 70 MΩ when filled with a 1 M KCl solution (Sutter P97 Brown Flaming type puller, Sutter Instruments, CA, USA). Electrodes were positioned by microdrives (Luigs-Neumann, Ratingen, Germany). The potential between the micropipette and the reference electrode (chlorided silver wire placed within the surgical wound close to the nerve) was 10x amplified and lowpass filtered at 10 kHz (SEC-05X, npi electronic GmbH, Tamm, Germany). Signals were digitized by a data acquisition board (PCI-6229, National Instruments, Austin TX, USA) at a sampling rate of 40 kHz (Signal No 1 in S1 Fig). Spikes were detected and identified online based on the peak-detection algorithm [43].

The EOD of the fish was measured between the head and tail via two carbon rod electrodes (11 cm long, 8 mm diameter, signal No 2 in S1 Fig, *Global EOD*). These electrodes were placed isopotential to the global stimulation to ensure the recording of the unperturbed fish field. The transdermal potential was estimated by a measurement close to the skin by a pair of silver wire electrodes, spaced 1 cm apart, which were placed orthogonal to the side of the fish just behind the operculum (signal No 3, *Reference EOD*). The measurement of the reference EOD includes the fish’s field as well as the stimulus and is thus a proxy of the signal driving the electroreceptors. A third pair of silver wire electrodes were mounted on the arm of a XYZ-robot (mph automation, Reutlingen, Germany, signal No 4 in S1 Fig, *Local EOD*) which could be moved alongside the fish.

EOD recordings were amplified by a factor between 100 and 1000 depending on the recorded fish’s EOD amplitude and bandpass filtered, cutoff frequencies of 3 Hz and 1.5 kHz for high and low-pass filter, respectively (DPA-2FXM, npi-electronics, Tamm, Germany).

Spike and EOD detection, stimulus generation and attenuation, as well as pre-analysis of the data were performed online during the experiment within the RELACS software version 0.9.7 using the efish plugin-set (www.relacs.net). Data was stored for further offline analysis in the open NIX data format (Resource ID: RRID:SCR_016196, https://github.com/g-node/nix, [44]). Datasets and all analysis code are available under the Creative Commons Attribution Non-Commercial Share-Alike 4.0 International License https://doi.org/10.12751/g-node.78xqwl.

### Stimulation

The fish were stimulated either with artificial electric fields mimicking the presence of other weakly electric fish or with random amplitude modulations. Stimuli could either be global or local. In both cases stimuli were attenuated (ATN-01M, npi-electronics, Tamm, Germany), and isolated from ground (ISO-02V, npi-electronics, Tamm, Germany). Global stimuli were delivered via two carbon rod electrodes (30 cm length, 8 mm diameter) placed on either side of the fish parallel to its longitudinal axis. Global stimuli were calibrated to evoke amplitude modulations of defined contrasts relative to the fish’s own unperturbed field measured close to the fish. Local stimuli were given using a pair of silver wire (0.25 mm diameter) electrodes mounted on the XYZ-robot (signal 6, S1 Fig). The silver wires were fixed within glass capillaries exposing only 1 mm tips. Local stimuli could not be calibrated to the respective local conditions but stimulus intensities were increased until a noticeable response modulation was observed in the recorded P-units (5–40 mV).

Random amplitude modulations (RAM) were created by multiplying the unperturbed global EOD with the desired noise waveform (frozen band-limited Gaussian noise, 0—300 Hz) and passing this signal into the experimental tank where it adds to fish’s EOD to result in the desired effect. The noise stimulus is calibrated to induce amplitude modulations with a standard deviation of 10% of the fish’s unperturbed field amplitude. RAM stimuli were always global.

### Data analysis

Offline analyses were performed with custom routines in python 3.8 using the following open source packages: scipy [45], numpy [46], matplotlib [47], nixio [44], and pandas [48].

### Estimation of receptor position

The position of a recorded electroreceptor afferent was estimated by applying a local stimulus that was moved along the rostro-caudal axis by means of a local dipole electrode mounted on a XYZ-robot (S1 Fig). The receptor position was estimated by fitting a Gaussian to the power at the expected frequency. The power spectrum was calculated from the time resolved firing rate of the neuronal response (see below) at any given stimulus position (S2 Fig). A segment length of 1 s was used with 50% overlap. A Hanning window was applied to each data segment and means were subtracted. Cells were recorded in the posterior branch of the lateral line nerve. We could therefore only record neurons with receptive fields on the trunk of the fish. The estimated receptive field positions are not affected by the stimulus intensity since the relative changes are important for localization and increasing the width of the tuning does not affect the position of the fitted Gaussian.

### Baseline analysis

**Fig 2:**
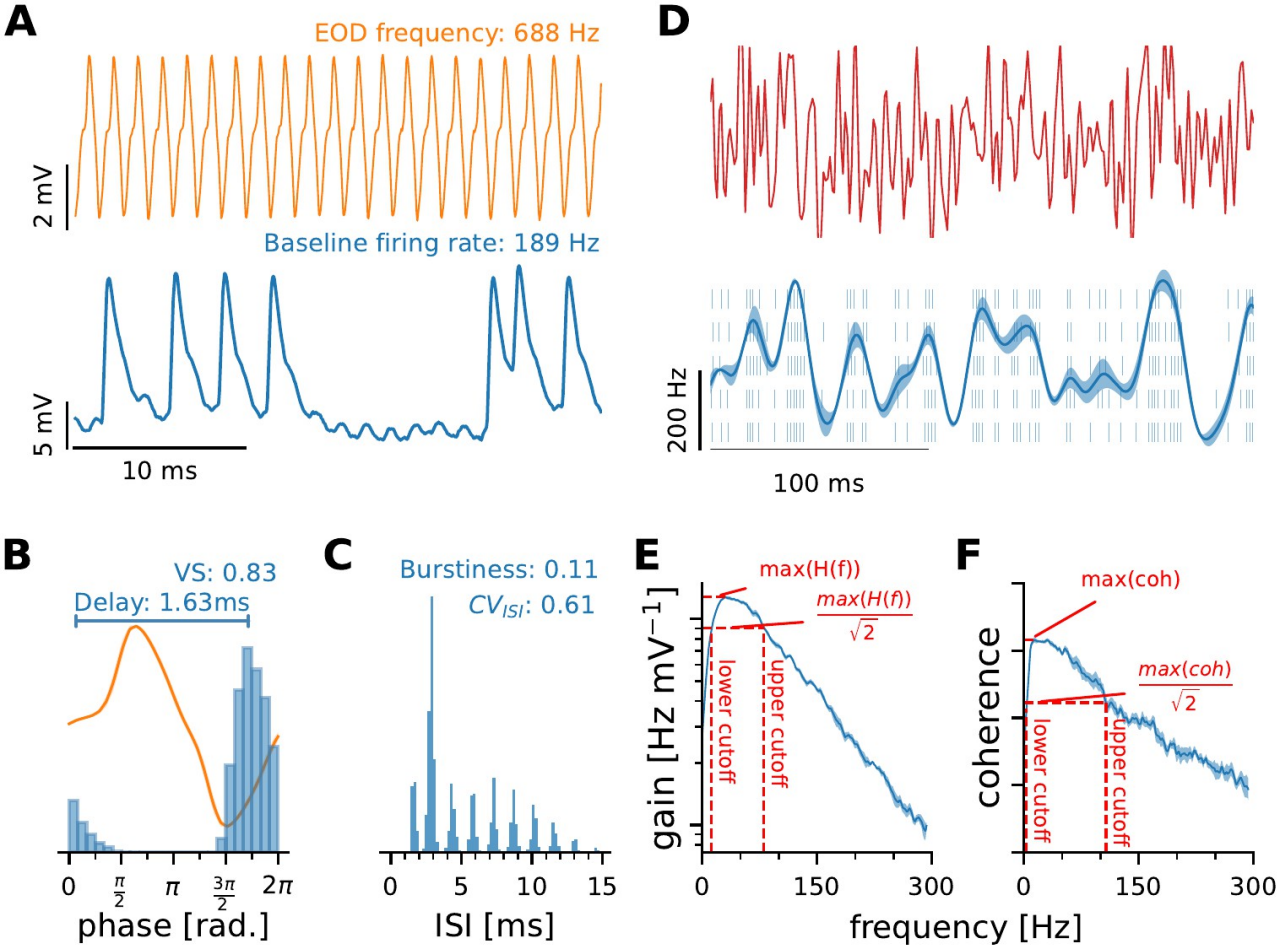
Analyzes on baseline and stimulus driven responses. **A—C**: Baseline response of an example P-unit. **A**: Short segments of the fish’s self-generated EOD (top) and the simultaneously recorded neuronal activity of a P-unit (bottom) in the absence of an external stimulus. This example neuron had a spontaneous activity of 189 Hz. **B**: Spike times are precisely timed within the EOD period. The average EOD waveform is depicted in orange and the blue histogram shows the phases in which action potentials occur. From this we calculated the vector strength (VS, Eq 1) which is 0.83 for this neuron. From the circular mean of the locking histogram we calculated the delay between EOD onset (time of the rising zero-crossing) and spiking activity. **C**: P-units skip EOD-cycles and sometimes fire bursts of action potentials. The interspike interval histogram shows the typical wide and multimodal distribution. Response regularity is characterized by the coefficient of variation (Eq 2, 0.61 in this example). The burstiness indicates the proportion of spikes occurring in intervals less than 1.5 times the EOD period [51] (0.11 in this example). **D:—F**: Driven responses of the same P-unit. **D**: The responses to frozen sequences of band-limited white noise of EOD amplitude modulations (0—300 Hz, top trace) are used to characterize the stimulus encoding properties. The stimulus profile is encoded in the spiking activity of the neuron (raster plot for 5 consecutive trials and firing rate in blue, bottom). The firing rate modulates around the baseline firing rate and the cell’s sensitivity is characterized by the strength of the modulation (Eq 4). The shaded area around the mean depicts the response variability, i.e. the across trial standard deviation of the firing rate. **E**: From the transfer function (Eq 6) we estimate the maximum gain as well as the upper and lower cutoff frequencies (red dashed vertical lines). **F**: Similar measures were extracted from the stimulus response coherence (Eq 7). The integral of the coherence spectrum is used a lower bound estimation of the mutual information between stimulus and response (Eq 8). The spectra in E and F have been smoothed with an 11 point running average. Abbreviations: VS, vector strength; CV, coefficient of variation; ISI, interspike interval; coh, coherence.

Each recording included several seconds of baseline activity in the absence of an external stimulus with only the fish’s own EOD being present (Fig 2A). Nevertheless, P-units show baseline firing that is highly irregular, sometimes bursty, but phase-locked to the EOD (Fig 2A, 2B and 2C). To characterize the spontaneous activity, the baseline firing rate, the vector strength, the coefficient of variation of the interspike-interval (*CV*_*ISI*_) distribution and the burstiness were quantified. Spike time locking to the EOD carrier was evaluated using the vector strength [49, 50].

$$\begin{array}{c} \rho f=|\sum_{j=1}^{n}\;\text{exp}\;(-i2\pi ft_{j})| \end{array}$$ (1)

with *n* the number of spikes, *t*_*j*_ the relative spike time of the j-th spike within the respective EOD cycle, and *i* the imaginary unit. The relative spike time is the delay between the spike time and the preceding rising threshold crossing at half-maximum of the EOD waveform. A vector strength of 0 indicates that spike phases are symmetrically distributed within the EOD period while a vector strength of 1 indicates perfect locking with all spikes occurring in the same phase of the EOD period.

The burstiness of the recorded cells was assessed using the burst fraction as proposed previously [51], i.e. the fraction of interspike intervals shorter than 1.5 times the average EOD period.

The coefficient of variation (*CV*_*ISI*_) is used to describe the response regularity [32, 33, 52]. A *CV*_*ISI*_ of zero indicates perfect regularity of the response, a value of unity is characteristic of Poisson firing. The *CV*_*ISI*_ is defined as:

$$\begin{array}{c} CV_{ISI}=\frac{\sigma_{ISI}}{\bar{ISI}}, \end{array}$$ (2)

with *σ*_*ISI*_ the standard deviation of the interspike-interval distribution and $\bar{ISI}$ the average interspike interval.

### Analysis of driven response

Characterization of the neuronal encoding of dynamic stimuli is based on the neuronal responses to band-limited white noise amplitude modulation stimuli (see stimulation, above; Fig 2D).

### Firing rate

The single trial firing rate, *y*_*k*_(*t*), was estimated by kernel convolution of spike responses with a Gaussian kernel

$$\begin{array}{c} F\left(t\right)=\frac{1}{\sqrt{2\pi \sigma_{gauss}^{2}}}e^{-\frac{t^{2}}{2\sigma_{gauss}^{2}}} \end{array}$$ (3)

with *σ*_*gauss*_ the standard deviation of the kernel which was 1 ms if not otherwise stated. The average firing rate (*y*(*t*)) is then calculated by averaging across trials.

### Estimating response modulation and variability

When driven by a stimulus the firing rate is modulated around the temporal average of the firing rate, 〈*y*(*t*)〉_*t*_, which is close to the baseline firing rate of the cell. The depth of the firing rate modulation is quantified by the standard deviation of the firing rate over time

$$\begin{array}{c} \sigma_{mod}=\sqrt{{\left\langle {\left(y\left(t\right)-{\left\langle y\left(t\right)\right\rangle }_{t}\right)}^{2}\right\rangle }_{t}}\;, \end{array}$$ (4)

where 〈⋅〉_*t*_ denotes averaging over time. The response modulation is used here as a proxy of the cell’s susceptibility for the stimulus.

The response variability was quantified by the standard deviation of the single-trial firing rates *y*_*k*_(*t*) across trials averaged over time:

$$\begin{array}{c} \sigma_{psth}={\left\langle \sqrt{{\left\langle {\left(y_{k}\left(t\right)-y\left(t\right)\right)}^{2}\right\rangle }_{k}}\;\right\rangle }_{t}\;, \end{array}$$ (5)

with *y*(*t*) the firing rate. Average firing rate and response variability are illustrated as solid line and shaded area in Fig 2D.

### Spectral analyses

In addition to time domain analyzes we calculated the transfer function (*H*(*f*), Fig 2E) according to:

$$\begin{array}{c} H\left(f\right)=\frac{\bar{P_{rs}}\left(f\right)}{\bar{P_{ss}}\left(f\right)}, \end{array}$$ (6)

where $\bar{P_{rs}}\left(f\right)$ is the average cross spectral density of response and stimulus and $\bar{P_{ss}}\left(f\right)$ the average power spectral density of the stimulus. Averaging was done across trials and segments of approximately 1 s duration (2^15^ samples) with 50% overlap. Segments were de-trended by subtracting the mean and a Hanning window was applied to each segment. From the transfer function we extracted the maximal gain as well as the lower and upper cutoff frequencies.

To estimate the (linear) information about the stimulus that is carried by the individual P-unit responses or by populations of P-units, the stimulus-response coherence ($\gamma_{sr}^{2}\left(f\right)$) was calculated

$$\begin{array}{c} \gamma_{sr}^{2}\left(f\right)=\frac{|\bar{P_{sr}}{\left(f\right)|}^{2}}{\bar{P_{rr}}\left(f\right)\bar{P_{ss}}\left(f\right)} \end{array}$$ (7)

where $|\bar{P_{sr}}\left(f\right)|$ is the absolute of the cross spectrum and $\bar{P_{rr}}\left(f\right)$ and $\bar{P_{ss}}\left(f\right)$ are the power spectra of the response and the stimulus, respectively. Averages are done across trials and segments. Spectra were estimated as described for the transfer function above. From the coherence spectrum we calculated the coherence rate, as a lower bound estimate of the mutual information between stimulus and response [53]

$$\begin{array}{c} MI=-\int_{0}^{f_{c}}\;{\text{log}}_{2}\;(1-\gamma_{sr}^{2}\left(f\right))\;df \end{array}$$ (8)

in which *f*_*c*_ is the cutoff frequency of the stimulus (300 Hz).

### Artificial populations of P-units

The information about the stimulus that is carried by neuronal responses was further estimated for artificial homogeneous and heterogeneous populations. Since we did single unit recordings these populations were constructed from neurons recorded in different animals in different recording sessions. In addition to the neurons recorded within this project we added 70 P-units recorded in a different project [33]. In total, the population analyses are thus based on 130 neurons recorded in 36 fish. All cells were stimulated with the same frozen Gaussian white noise AM stimulus.

From each neuron a set of up to 10 homogeneous populations was created for population sizes ranging from 1 to the number of trials recorded in that particular neuron. Population responses were assembled from unique random combinations of trials. The population response is the average firing rate estimated with a Gaussian kernel of 1.0 ms standard deviation. From each population response the response modulation (Eq 4) and the average firing rate were extracted. Additionally, the stimulus response coherence was estimated (Eq 7) and the mutual information (Eq 8) as well as the lower and upper cutoff frequencies of the coherence spectrum were estimated.

Heterogeneous populations were created by randomly drawing trials from all recorded neurons. Per population each cell could contribute with at maximum one trial. For each population size (2 to 30 neurons) 50 different populations were created. Each selected trial was then temporally aligned with the stimulus by subtracting the average delay (estimated from the peak position of the spike-triggered average stimulus) from the recorded spike times. The population response was estimated as the average PSTH across trials. When artificial delays were added, all spikes of an individual trial were shifted by the same delay that was drawn from a Gaussian distribution with zero mean and the respective standard deviation (*σ*_*delay*_) after the response was temporally aligned to the stimulus as described above.

### Leaky integrate and fire model

Leaky Integrate and Fire (LIF) [42] model neurons were created by numerically solving

$$\begin{array}{c} \tau_{m}\frac{dV}{dt}=-V+I_{offset}+I_{stimulus}+\sqrt{2D}\xi \left(t\right) \end{array}$$ (9)

in which *τ*_*m*_ is the membrane time constant, *V* the membrane voltage, *I*_*offset*_ an offset current, *I*_*stimulus*_ the stimulus current and an additional noise current drawn from a Gaussian distribution *ξ*. Δ*t* is the temporal step size of the simulation. Whenever the membrane voltage exceeded the threshold *θ*, the spike time was noted, and the membrane voltage was reset to *V*_*reset*_.

The population was modeled as a 1-dimensional array of LIF model neurons with identical parameterization and independent noise. On the one-dimensional array spacing of neurons was assumed to be equal. All model neurons were driven with the same 2 s frozen white noise sequences which had spectral power in the ranges 0—100 Hz, 100—200 Hz, or 200—300 Hz created from Gaussian white noise (strength of the stimulus is controlled via the standard deviation, *σ*_*stimulus*_). All model parameters are listed in Table 1. The population response was estimated as the average firing rate estimated by kernel convolution as described above using a Gaussian kernel with a standard deviation of 1.25 ms.

**Table 1:** Model parameterization. Parameters in the membrane model part refer to Eq 9.

Membrane model						Population model	
*τ*_*m*_	*I*_*offset*_	$\sqrt{2D}$	*θ*	*V*_*reset*_	Δ_*t*_	*σ*_*stimulus*_	cell density
0.25 ms	2.5	5	1	0	0.01 ms	0.0625	2000 m^−1^

## Results

A total of 84 p-type electroreceptor afferents were recorded in 13 subjects of *Apteronotus leptorhynchus* to estimate the population heterogeneity and its potential dependency on the receptor’s position. In each cell we recorded the baseline activity and, in a subset, the responses to dynamically varying sequences of frozen band-limited white noise amplitude modulations. From baseline and driven responses we extracted several features to characterize the cells (Fig 2).

### Most baseline parameters are not correlated with receptive field position

**Fig 3:**
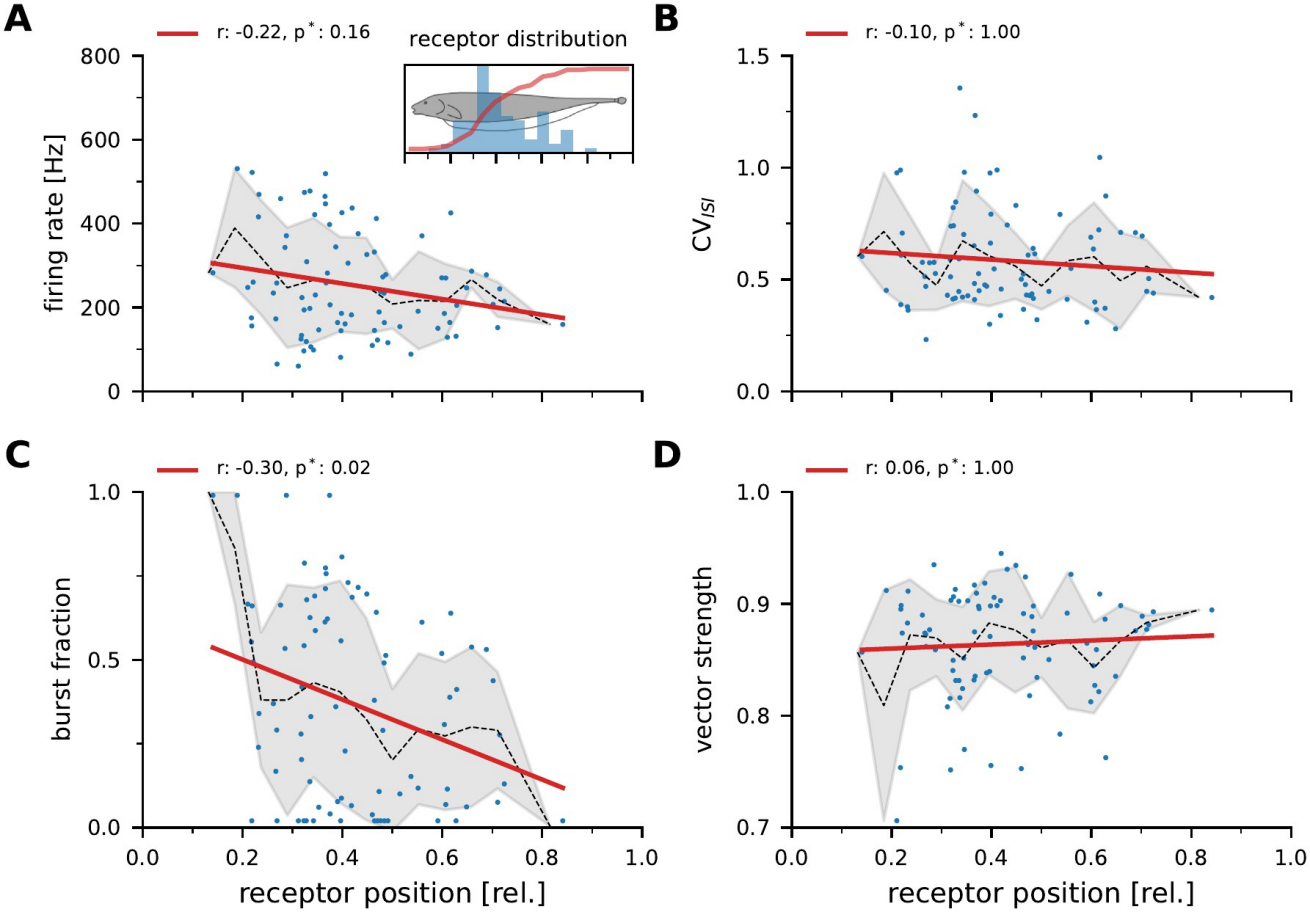
Correlation of baseline response properties and receptor position. r-values are the Pearson correlation coefficients, p-values are Bonferroni corrected. Dashed thin line shows the mean and gray areas the standard deviation estimated in bins of 10% width. **A**: Baseline firing rates estimated with only the unperturbed fish’s field present. Inset: In this study 84 p-type electroreceptor afferents were recorded. The blue histogram in the inset shows the distribution of receptor positions along the rostro-caudal body axis expressed relative to the total body length. The cumulative (red line) indicates that three fourths of the recorded cells were recorded in the frontal third of the body. Due to the recording location cells in the frontal 20% of the body could not be recorded. **B**: Coefficient of variation of the interspike interval distribution (CV_ISI_) during baseline activity. **C**: Fraction of action potentials observed as part of action potential bursts as previously defined [51]. **D**: Vector strength quantifying the phase-locking to the EOD.

The majority of the recorded P-units had receptive field positions between 30 and 50% of the total body length (Fig 3A, inset). In our sample no cells with receptive field positions beyond 80% of the body length could be recorded. Cells more frontal than about 20% of the body length could not be recorded from, given the recording location in the posterior branch of the lateral line nerve (see Methods). In the absence of an external stimulus (but in the presence of the unperturbed own electric field) P-units show a spontaneous baseline activity (Fig 2A, 2B and 2C) that is very heterogeneous across cells, even when recorded in the same animal [32, 33]. From the baseline activity we extracted five characteristics: the baseline firing rate, the coefficient of variation of the interspike intervals (*CV*_*ISI*_), the fraction of spikes that occurred in bursts of action potentials and the locking of the spike times to the EOD (see Methods, Fig 2A, 2B and 2C). These features were then correlated with the estimated receptor position (Fig 3A, 3B, 3C and 3D). The overall range of observed firing rates varies between about 60 and 530 Hz (249±117 Hz) matching previously published observations [32, 33]. The *CV*_*ISI*_ (0.52±0.19), the burst fraction (0.3±0.26), and the vector strength (0.86±0.05) fall well within the ranges reported before. The firing rate, *CV*_*ISI*_, and vector strength do not statistically significantly depend on the receptor’s position (Fig 3A, 3B and 3D). The burst fraction shows a statistically significant decline in our sample (r=-0.30, p = 0.02). The vast spread of the data and the few points at the most frontal and most caudal positions may bias this observation.

### Coding properties are not correlated with receptive field position

**Fig 4:**
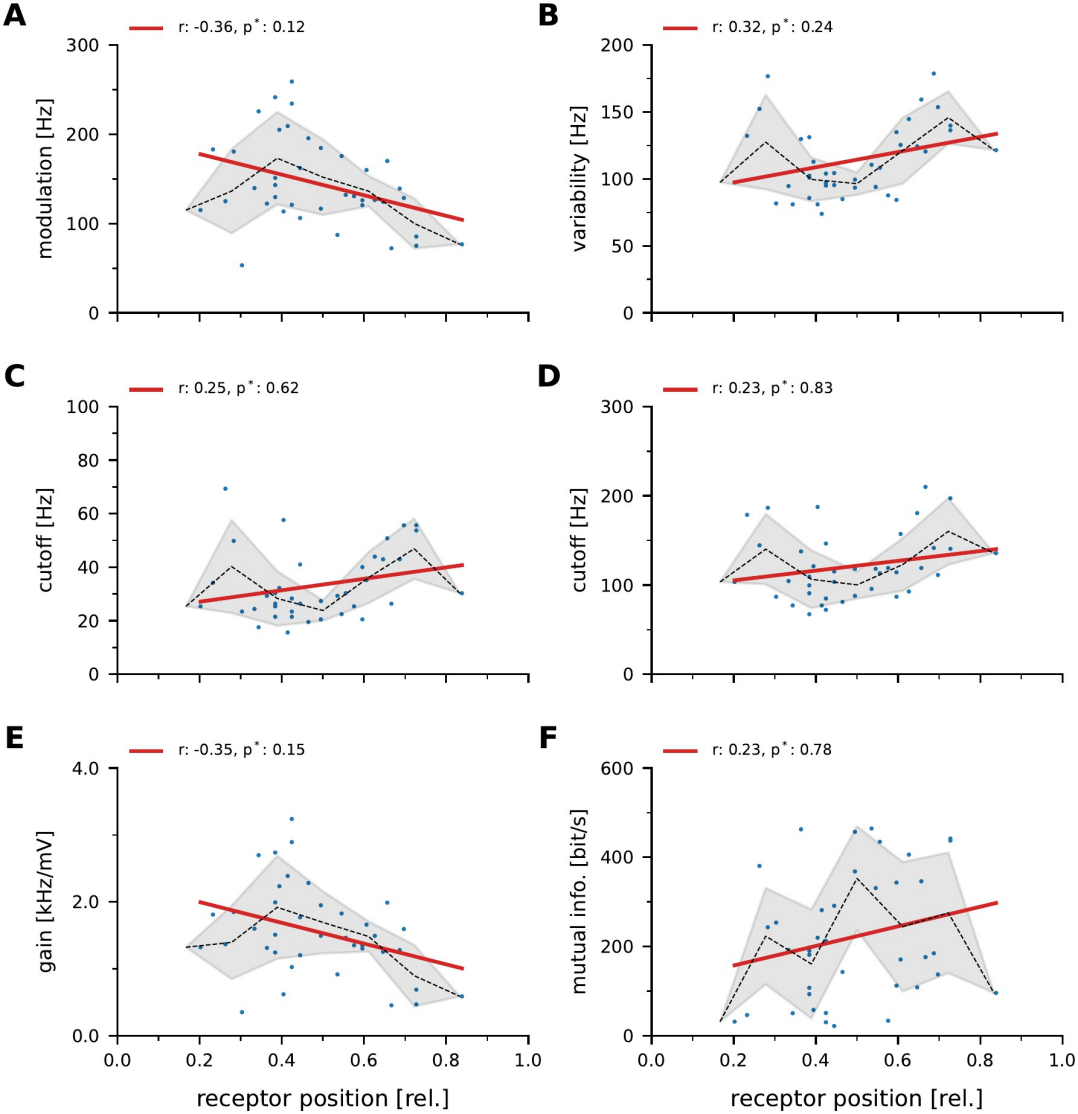
Correlation of stimulus driven response properties and receptor position. r-values are the Pearson correlation coefficients, p-values are Bonferroni corrected. Dashed thin line shows the mean and gray areas the standard deviation estimated in bins of 10% width.**A**: Response modulation of the across-trial average firing rate (Eq 4) in response to frozen white noise amplitude modulations. Please note that these stimuli were only presented to a subset of 39 neurons which could be recorded sufficiently long to allow for position estimation and stimulation with white noise stimuli and were stimulated with the same stimulus intensity (10% contrast). **B**: response variability, i.e. the across-trial standard deviation of the firing rate (Eq 5). **C, D**: lower and upper -3 dB cutoff frequencies of the transfer function. **E**: peak gain of transfer function. **F**: Mutual information between responses and the stimulus estimated from the stimulus response coherence spectrum.

In a subset of 39 neurons recording durations allowed for analyzing the stimulus encoding performance using sequences of white noise amplitude modulations of the fish’s field (Fig 2D–2F, see Methods). The cellular responses were characterized by the response modulation of the across-trial average firing rate (Eq 4, Fig 4A, 145 ±49 Hz), the across-trial response variability (Eq 5, Fig 4B, 114±27 Hz), and by using the transfer function to characterize the spectral coding range. From the transfer function we extracted the lower and upper cutoff frequency (33±13 Hz and 121±37 Hz, respectively), and the maximum gain (1553 ±32 Hz/mV, Fig 4C, 4D and 4E, respectively). From the coherence spectrum we quantified the amount of information carried by the responses about the stimulus by estimating the lower-bound mutual information as the integral of the coherence spectrum (Eqs 7 and 8, [53]). In the population of recorded cells the mutual information varies strongly (Fig 4F, median: 181 bit/s, interquartile range from 106 to 286 bit/s). All measures of the stimulus encoding performance vary considerably across cells, but none showed a statistically significant correlation with the receptor location. Many baseline and driven response properties show correlations. For example, the average firing rate is positively correlated with the rate modulation of the driven response which, in turn is a good predictor for the amount of mutual information about the stimulus that carried by the neuronal response (S3 Fig).

### Receptive field position determines preferred phase and response delay

The vector strengths depicted in Figs 2B and 3D show the tight temporal coupling between P-unit spikes and the EOD period [50, 54]. According to the expectation shown in Fig 1, the delay between the globally measured EOD and the spike time should be influenced by the receptor position and the axonal conduction velocity.

**Fig 5:**
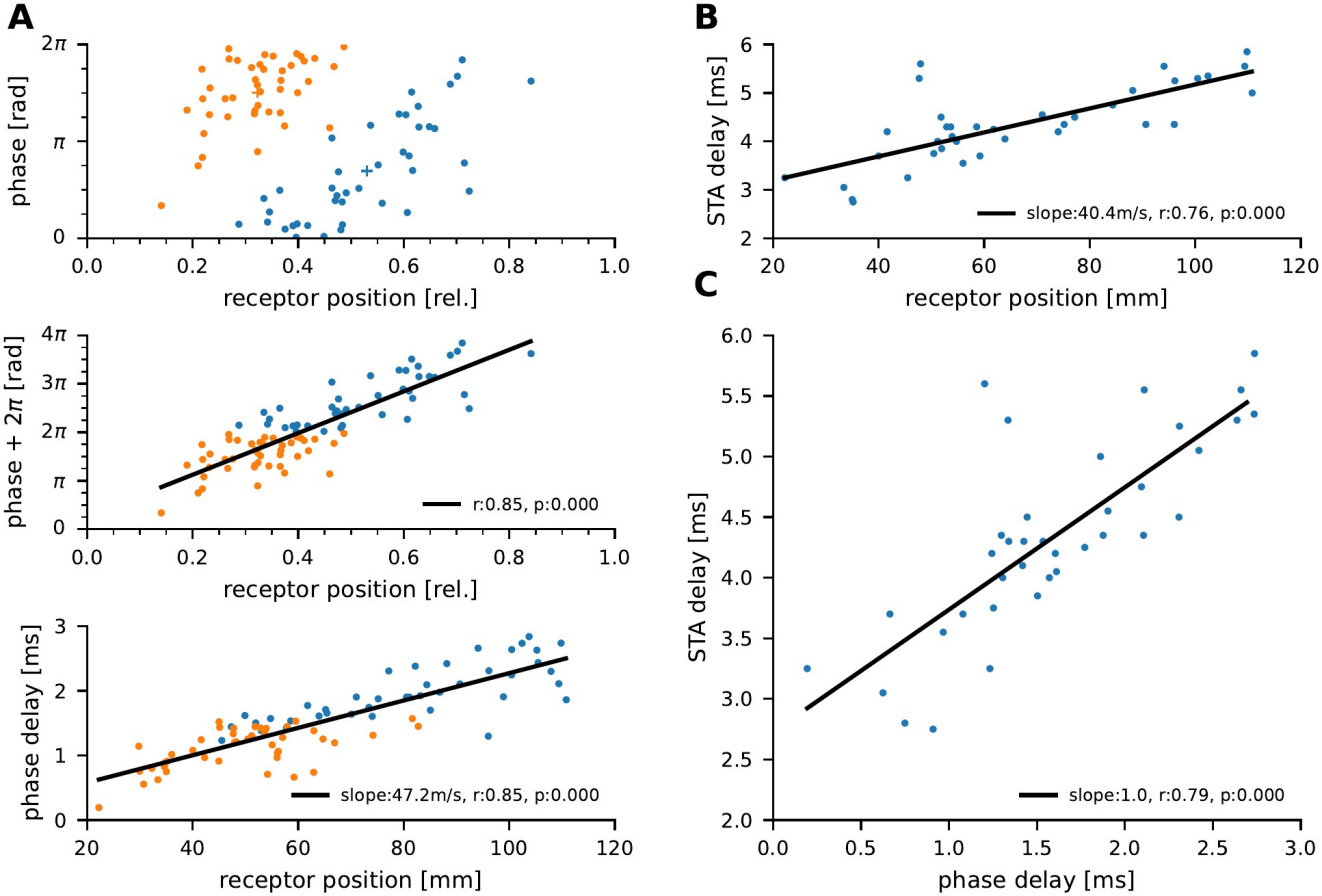
Preferred phase and response delay depend on receptor position. **A**: Top: Phase relation between the baseline spikes and the fish’s own EOD measured at the operculum (*Reference EOD*, S1 Fig). Colors represent clusters as established by K-means clustering. Crosses depict cluster centroids. Middle: Correlation of preferred phase and receptor position after the caudal (blue) cluster was shifted by one EOD cycle (2*π*). Now phases correlate positively with the rostro-caudal receptor position (solid line, Pearson correlation). Bottom: Re-plotting of the data in absolute units. Spike phases are expressed as the absolute delay between the beginning of the EOD (ascending zero crossing) and the respective action potential. The receptor positions on the rostro-caudal axis are given in absolute numbers relative to the snout position. The slope of the regression line is the inverse of the conduction velocity of 47.2$\frac{m}{s}$. **B**: Delay between the cellular response and the white noise stimulus estimated from the peak of spike triggered average stimulus (STA) for those cells in which white noise responses could be recorded (n = 39). **C**: Correlation of the STA delay and the absolute phase delay.

The effect of receptor position on the response delay was estimated based on the baseline activity in all recorded neurons (n = 84) Fig 5A. On first glance, the phase relation between the baseline spikes and the EOD appears independent of the receptor position. K-means clustering of the data reveals two clusters and phase shifting the caudal cluster by one EOD cycle (2*π*) shows that the preferred phase within the EOD-cycle indeed depends on the receptor position and can be well fitted with a linear regression (r = 0.85, p < 0.01). The inverse of the slope of the linear regression is then the conduction velocity (47.2*m*/*s*).

In the subset of cells in which we could record responses to band-limited white noise stimuli, we also estimated the response delay from the peak position of the spike triggered average stimulus (STA, n = 39, Fig 5B). Similarly, the STA delay depends on the receptor position (r = 0.76, p < 0.01) the conduction velocity, however, estimated using this approach based on the subset of cells (n = 39) is 40.4*m*/*s*. Phase delay between spikes and EOD and STA delay are in very good agreement within the same cells (Fig 5C).

### Pyramidal cells in the ELL might gain from population heterogeneity in their input layer

Postsynaptic neurons in the ELL receive information from up to about 1000 P-units [39]. Since most of the baseline as well as the stimulus encoding properties of the P-units do not depend on the location on the body (except for the preferred phase), each pyramidal neuron will integrate the activity of a similarly heterogeneous population of P-units.

To test whether stimulus encoding in pyramidal neurons profits from P-unit heterogeneity, we constructed artificial homogeneous or heterogeneous populations from the pool of recorded P-units (see also S4 Fig). All cells were stimulated with the same white noise stimulus of amplitude modulations which has spectral power in the range 0—300 Hz. For the following analyzes we extended our dataset to 130 cells by including 70 cells from a previous study [33] for which no receptor location was estimated (60 in total from this study).

**Fig 6:**
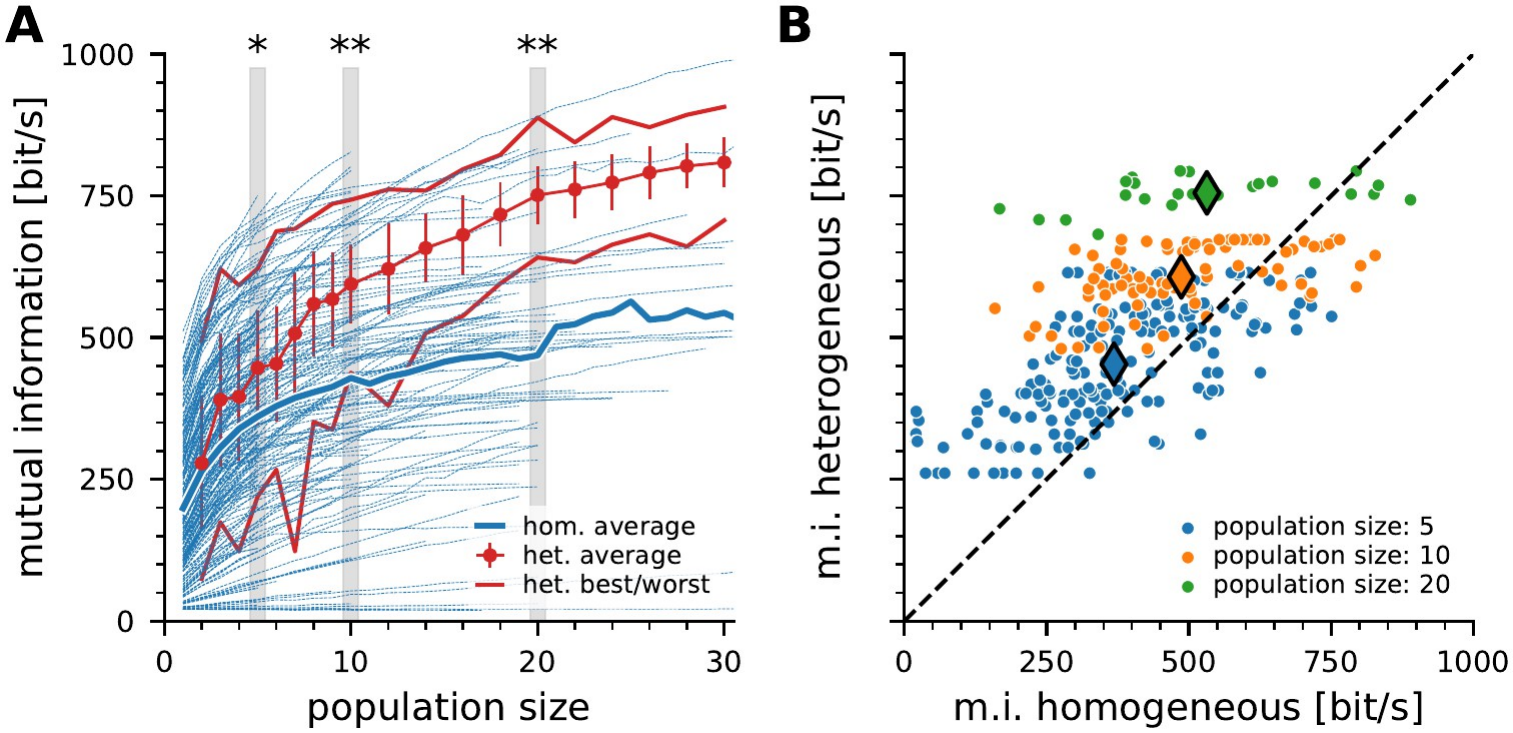
Information content of homogeneous and heterogeneous populations of P-units. **A**: Mutual information estimated from the stimulus response coherence (Eq 8) carried by populations of increasing size. Blue lines show information content of homogeneous populations created by combining randomly chosen trials of the same neuron. Since not all recordings have the same number of stimulus repetitions, the maximum possible population size varies between recorded neurons. Thick blue line is the average across all homogeneous populations. Red dots show the average information estimated from heterogeneous populations (see Methods for details). Error bars indicate the standard deviation, thin red lines depict the best and worst performance observed in the sample of heterogeneous populations. Tests on statistically significant differences in mutual information in homogeneous and heterogeneous population were performed using a Mann Whitney U-Test with Bonferroni correction for the highlighted population sizes, one asterisk: *α* < 5% two stars indicate *α* < 1%. **B**: Comparison of the mutual information carried by homogeneous (x-axis) and heterogeneous (y-axis) populations that are similarly driven by the stimulus (response modulation of heterogeneous population response within a ±20% range of the respective homogeneous response) for three different population sizes. Large symbols depict the center of gravity of the respective distributions. Dashed line is the identity line. Abbreviations: m.i., mutual information; het., heterogeneous; hom., homogeneous.

Homogeneous populations were constructed by combining responses recorded in the same neuron. Depending on the recording duration the number of recorded trials varies across cells and therefore the maximum population size varies accordingly. With an increasing population size the mutual information between the population response and the stimulus increases (blue lines in Fig 6A). On average, the amount of information carried by the homogeneous populations roughly doubles from 1 to 30 cells (thick blue line, 261 bit/s with two cells to 536 bit/s with 30 cells).

Heterogeneous populations were constructed from combinations of responses from different cells that may even be recorded in different animals (see Methods for details). The population response was calculated after the individual responses were temporally aligned with the stimulus which eliminates variations in delays. As for homogeneous populations the information carried by heterogeneous populations increases with population size (295 bit/s with 2 cells to 704 bit/s with 30 cells). The average heterogeneous population carries more information about the stimulus than the average homogeneous population. The best heterogeneous population does not exceed the performance of the best homogeneous populations. But, conversely, the worst heterogeneous population performs generally better than many homogeneous populations (Fig 6A, red dots, thin red lines for best and worst heterogeneous population). The information gain through heterogeneity is statistically significant for populations larger than four cells (tested for the highlighted population sizes in Fig 6A). In general, the firing rate modulation is a good predictor of the mutual information (S3 Fig). For a fair comparison we compare the coding performance of homogeneous and heterogeneous populations that show similar (± 20%) response modulations of the population response (Fig 6B). For populations larger than five cells, the vast majority of heterogeneous populations carries more information than the respective homogeneous ones. The center of gravity deviates increasingly from the identity line (large diamond markers) for larger populations.

### Neuronal conduction delay acts as an information filter in the population response

In the population analysis described above the responses were aligned to the stimulus and thus every spike contributing to the population had the same delay. This is not the case in real populations when axon lengths vary depending on their origin within the receptive field.

The heterogeneous populations were now used to systematically study the effect of conduction delays on the stimulus encoding performance. As above, responses were aligned to the stimulus and then an artificial delay (drawn from a Gaussian normal distribution with a standard deviation, *σ*_*delay*_) was added to the spikes. The same delay was applied to all spikes originating from the same neuron to mimic conduction delays and not to increase spike time jitter.

**Fig 7:**
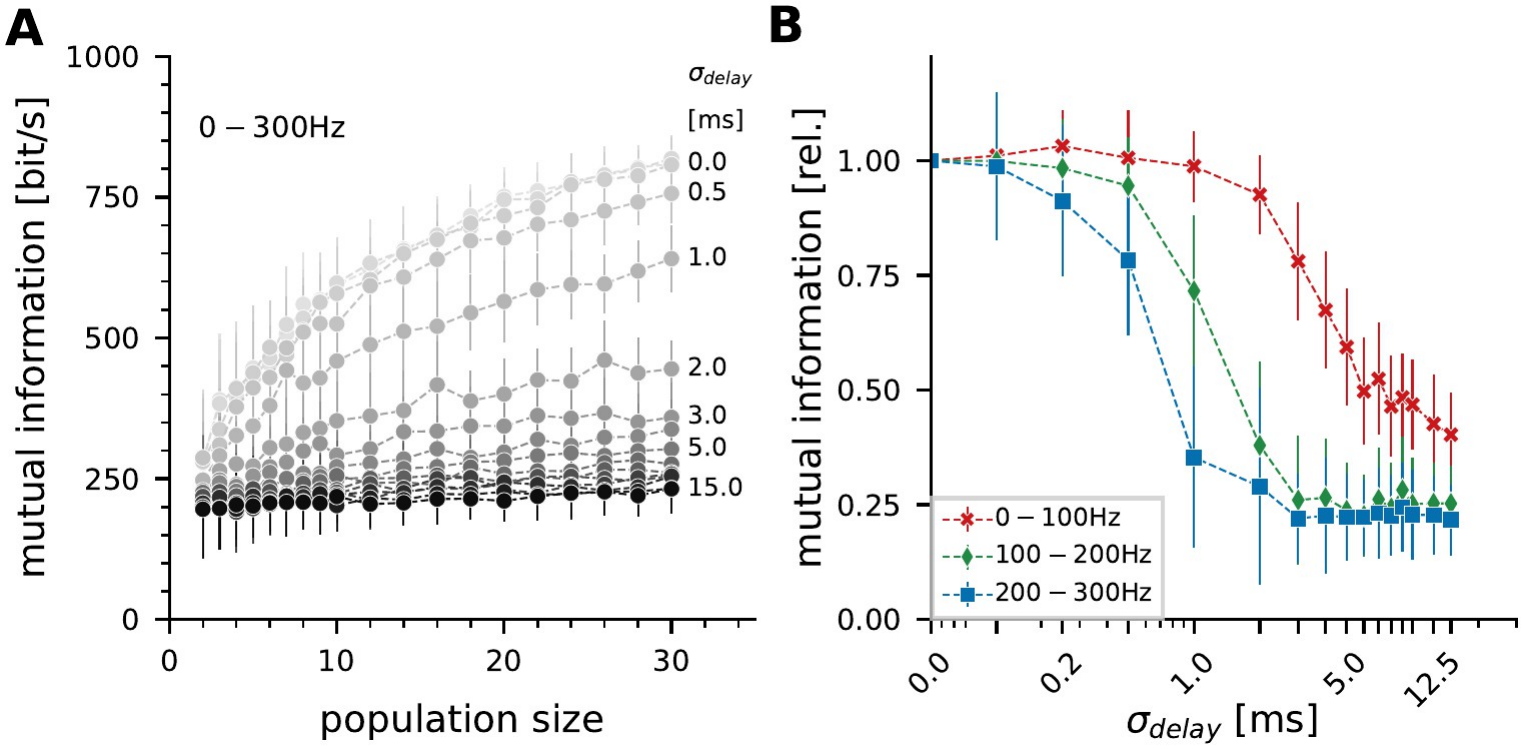
Conduction delays in a population act as an information filter. **A**: Average mutual information carried by heterogeneous populations of increasing population size that are subjected to varying simulated conduction delays. **B**: Mutual information as a function of conduction delay compared for the three different spectral bands. High frequency information is more strongly affected than low-frequency content. Mutual information is normalized to the zero-delay performance, a population size of 20 neurons was chosen here.

With increasing *σ*_*delay*_ the amount of information drops. The larger the delay, the more severe is the effect. In the extreme, there is hardly any gain through population averaging (Fig 7A, increasing *σ*_*delay*_ from light to dark color). When separating the stimulus encoding performance for three different spectral bands it becomes clear that the deteriorative effect is not equally strong for all stimulus components (Fig 7B, population size of 20 neurons). Low-frequency components are more robust against delays in the population than high-frequency components. Independent of the added delay, P-unit populations encode higher frequencies worse than lower spectral bands (compare also to the transfer or coherence spectra shown in Fig 2E and 2F). Encoding high-frequency changes in the stimulus requires higher precision in the spike times than encoding slow changes and is thus more sensitive to the spread of delays across the population.

### Conduction delays and stimulus dynamics constrain meaningful population sizes in populations of model neurons

The results shown above indicate that the amount of information conveyed by populations of P-units is affected not only by the population size and the noise in the system but also the spread of conduction delays within the population. In particular, high-frequency information suffers from a too large spread of conduction delays. Assuming a constant conduction velocity and a homogeneous receptor density across the sensory surface, this is directly related to the spatial extent of the population. To extend these ideas beyond the electric fish, we performed simulations using a leaky integrate and fire (LIF) models with independent noise for each model neuron. The model neurons were not fitted to any realistic neuron (for details on the model parameterization see Methods). The model cells were stimulated with band-limited white noise stimuli which had spectral power in three different ranges (0—100 Hz, 100— 200 Hz, and 200—300 Hz) and project to an integrating neuron via axons with three different conduction velocities (7 m/s as observed in visual fibers in the monkey corpus callosum [55], 25 m/s as a representative for the squid giant axon [18], and 50 m/s as estimated here for the p-type electroreceptor afferents). An arbitrary cell density of 2000 *m*^−1^ was assumed. The mutual information between the population response and the stimulus was again estimated from the stimulus-response coherence.

**Fig 8:**
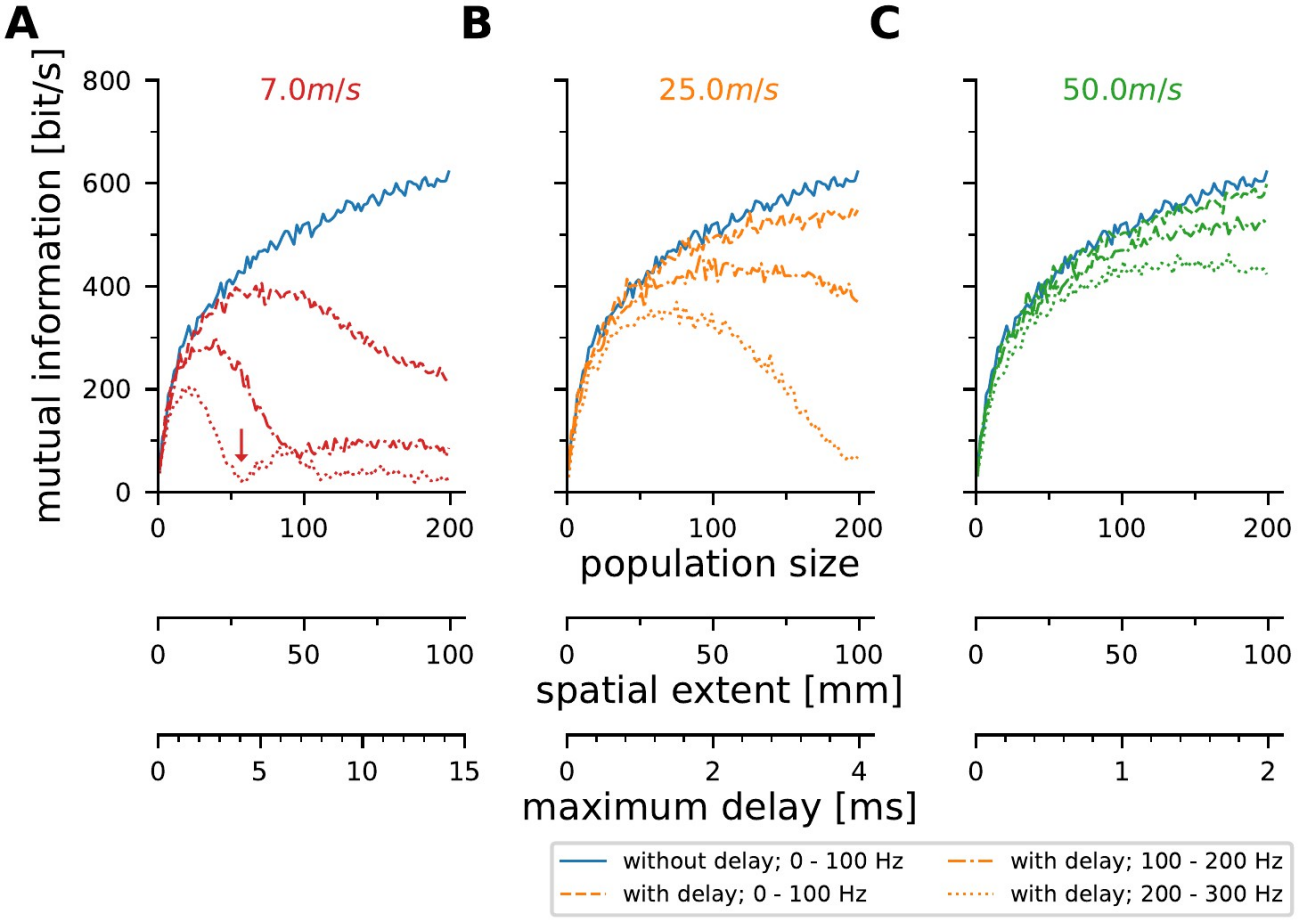
Conduction velocity and stimulus dynamics constrain meaningful population sizes of LIF model neurons. **A—C** Mutual information between stimulus and response estimated from the population responses of LIF-model neurons (see sketches in Fig 1A). These models assume 1D populations with a density of 2000 neurons per m of the sensory surface. Model parameterization was completely arbitrary and identical for all model neurons (see Methods). The models were driven with the same frozen noise sequences with spectral power in the ranges 0–100 Hz, 100–200 Hz and 200–300 Hz (dashed, dash-dotted, and dotted lines, respectively). Each model had the same amount of (independent) noise added to the driving stimulus. Three realistic conduction velocities were simulated and compared to the unrealistic instantaneous conduction (blue solid line in all figures, “without delay”). With decreasing conduction velocity the encoding performance drops at smaller populations. High-frequency encoding is most sensitive to spread in conduction delays. The “harmonic” structure seen in A is a consequence of the stimulus’ auto-correlation. **A**: Conduction velocity of 7 *ms*^−1^, as described for some visual fibers in the monkey corpus callosum [55]**B**: 25 *ms*^−1^ for example measured in squid giant axons [18], **C**: 50 *ms*^−1^ as estimated above for P-unit afferents.

Increasing the population size increases the mutual information if no conduction delay is assumed (blue solid lines in Fig 8A, 8B and 8C). When considering realistic conduction velocities, the arising spread of conduction delays within the population (see Fig 1) affects the amount of information carried by the population response. In line with the previous experimental observations, the severity depends on the stimulus dynamics and the conduction velocity (broken, dash-dotted and dotted lines representing different stimulus dynamics in Fig 8). At low conduction velocities, the limitations imposed by delays become more severe. Increasing the neuronal population size at a conduction velocity of 7 m/s initially increases mutual information but at a certain population size becomes detrimental (Fig 8A). For high-frequency stimuli a population of 57 neurons carries as little information about the stimulus as a single neuron does (Fig 8A, arrow). In the simulations performed here the population size is directly interchangable with the spatial extent of the population on e.g. the sensory surface and the resulting conduction delays. As indicated above, these observations depend on the stimulus dynamics. If the population is meant to encode only slowly varying stimuli larger delays can be tolerated and thus larger populations may be beneficial. The opposite is true if the population is meant to faithfully encode high-frequency stimuli. Then faster conduction or a more densely packed population is required to keep the maximum delays low.

## Discussion

Here we studied the heterogeneity among p-type electroreceptor afferents in *A. leptorhynchus*. The presented data confirms previous observations of the large spread of baseline and stimulus encoding properties in P-units [17, 32, 33, 52]. Only minor parts of the observed variance can be explained by the receptor location. Even for the burst fraction, the only parameter found to statistically significantly depend on location, only 9% of the total variation can be attributed to the position (Fig 3C). The spatial homogeneity in P-unit heterogeneity implies that postsynaptic neurons in the hindbrain may always receive heterogeneous inputs from the sensory periphery—regardless of their location. The system can profit from the heterogeneity since, on average, heterogeneous populations carry more information about the stimulus than the average homogeneous population of the same size. Generally, mutual information increases with population size but integrating over a spatially large patch of the sensory surface unavoidably leads to a spread of temporal delays within the population. This is an effective filter on the information content and particularly critical for high-frequency stimulus components. An effect that is not fish-specific but is also observed in populations of leaky integrate-and-fire model neurons.

### Origin of heterogeneity in P-units is unclear

Previous work shows that the tuning of newly generating electroreceptor organs is off for a few weeks [56], these organs are usually smaller with fewer primary electroreceptors per organ and there is variability in the number of electroreceptor organs innervated by a single afferent fiber [57]. Such variations may have consequences for the sensitivity and the response reliability of the P-unit in total. Geometrical effects due to the curved body surface [58, 59] and the fact that the electric field intensity is not uniform over the body surface [26, 60] will have an impact on the sensitivity as well but do not explain the heterogeneity in other parameters.

### P-unit heterogeneity beneficial in more than one context

When two fish interact, the interference of their fields not only leads to regular amplitude modulation (AM) but, as the fish move relative to each other, the strength of the AM will change as a function of time [61–63]. Extracting these so-called envelopes requires non-linear meachanisms [17, 61]. Savard et al. [17] concluded that the heterogeneity among P-units makes sure, that there are always cells with sufficiently high or low firing rates that show saturation non-linearities which then enable the P-units to encode envelopes. Our population analyzes show that heterogeneity is beneficial for the encoding performance even when we only consider linear encoding (as the stimulus response coherence is a linear method). We would argue that even if some cells are saturated by the stimulus, others in the population will still be able to encode changes in stimulus amplitude. Thus, the interpretations are not mutually exclusive but hint at heterogeneity being beneficial in more than one context.

### Conduction delays explain location dependent phase shift

P-units are known to fire action potentials tightly phase-locked to the EOD which can be characterized by the vector strength [26, 49, 50, 64] (Fig 2B). The preferred phase of action potential firing within the EOD period (Fig 5A) shows a strong dependency on receptor location. The convincing linear correlation suggests that the delays can be fully explained by the distance between receptor location and recording site assuming a constant axonal conduction velocity. The conduction velocity was estimated to 47.2 m/s which is faster than described for other Gymnotiform electric fish: For *Eigenmannia virescens* conduction velocities of 30 *m*/*s* for receptors on the trunk and 15 m/s for afferents on the head were reported [65]. For *Hypopomus sp*. speeds of 25 m/s were reported but this study does not describe differences between frontal and caudal receptors [58]. Soma volumes of tuberous afferents in *E. virescens* were shown to be larger for more distant receptors and a correlation between soma volume and axon diameter and thus conduction velocity was suggested [65]. Our data does not show such dependence and also the original data seems to be well-fitted by a linear regression [65]. In contrast to the previous works who used steps in the replacement fields to estimate the delay between stimulus onset and the response we here used the zero-crossings of the head-to-tail measurement of the EOD as a reference. The EOD waveform is not the same all over rostro-caudal body extent. Rather, it changes in amplitude and waveform [60, 66] and in particular in the center region of the fish the 2nd harmonic of the EOD frequency becomes dominant and induces a phase shift of the zero-crossings. The P-units in these regions might therefore respond in a different phase relation to the global head-to-tail measurement. These effects might induce a phase advance of about 1/2 of the EOD-period, i.e. between 0.5 and 0.8 ms. In our data, however we do not see any obvious effects of these phase shifts. P-units fire probabalistically to the EOD cycles. They may skip the “first” period of the 2nd harmonic and then fire at the “second” which would then average out. In a second approach of conductance delay estimation we used the subset of cells that were stimulated with white-noise stimuli. There, the delay of the spike-triggered average stimulus and the spike time shows the same dependency, though with a shallower slope, (Fig 5B) and is consistent with our head-to-tail delay estimation (Fig 5C).

Conduction velocity depends on the axonal morphology. Increasing it is an energetically expensive investment [19]. While both, *E. virescens* and *A. leptorhynchus* are wave-type electric fish, their EOD frequencies occupy very different ranges, 250 to 450 Hz in *E. virescens* and 600 to 1000 Hz in *A. leptorhynchus* [67, 68]. This higher EOD frequency in *A. leptorhynchus* enables it to encode a wider signal bandwidth (as predicted from the sampling theorem, see also S3 Fig), but in order to preserve the additional information higher conduction velocities are needed. From the *E. virescens* perspective investing in faster conduction may not pay off if the stimulus dynamics are limited to lower frequencies for other reasons.

### Conduction delay acts as information filter

**Fig 9:**
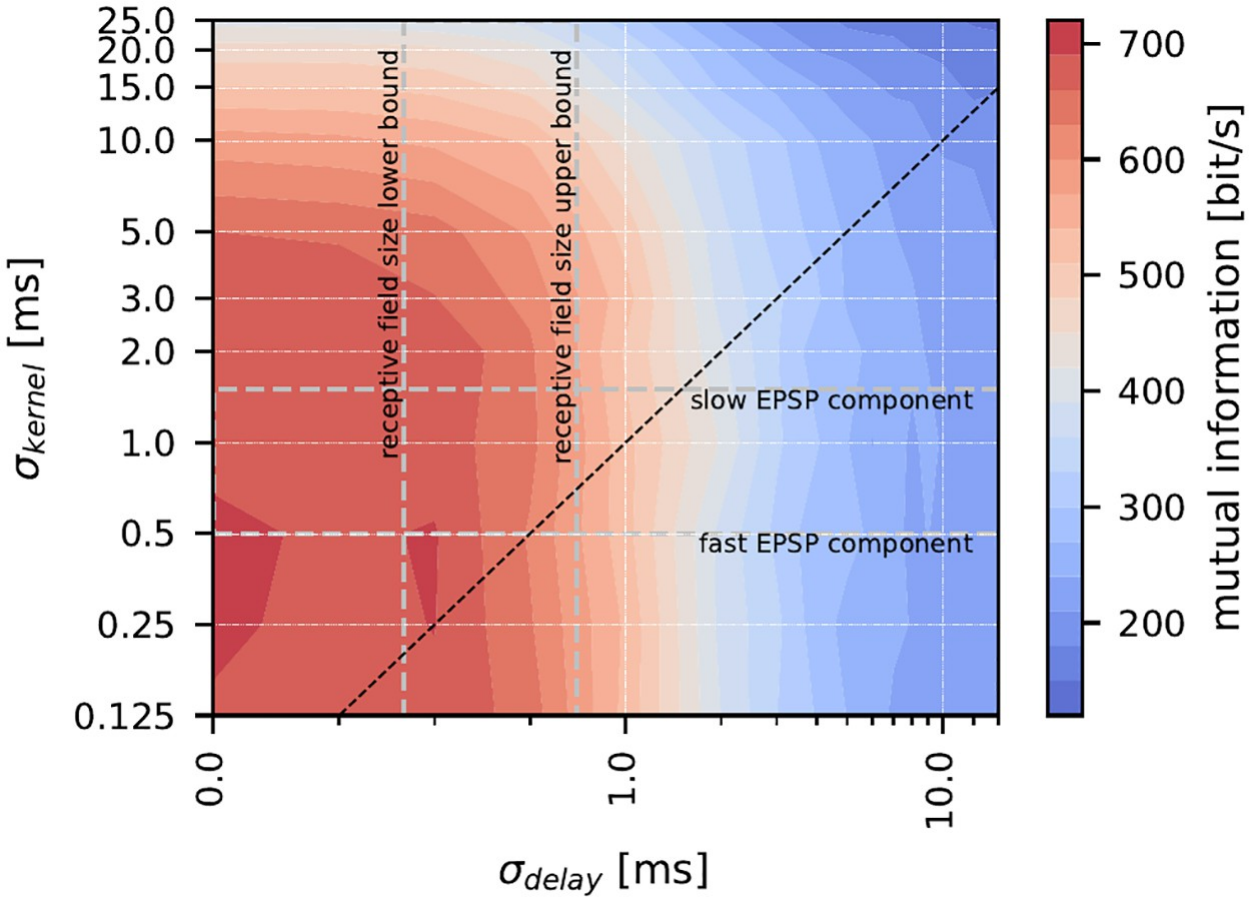
Information filtering by conduction delays is stronger than by synaptic filtering. Comparison of the effects of conduction delays (x-axis) and synaptic transfer (y-axis) on the information content of the population response (color code). Dashed black line is the identity line. Area between horizontal dashed lines depicts the standard deviations (*σ*_*kernel*_) of the fast and slow EPSP components as reported for the P-unit to pyramidal cell synapses [70]. Vertical dashed lines enclose the range of conduction delays that can be expected for pyramidal neurons in the lateral segment of the electrosensory lateral line lobe [39] considering the 2*σ* or 4*σ* receptive field sizes. The data shown here assumes a population size of 16 cells.

The spread of conduction delays within populations strongly affects the information carried by the population response. Delays act much like a low-pass filter, attenuating high-frequency content more effectively than low-frequency content. Representing high-frequency stimuli requires high spike timing precision. Differences in spike arrival from the two ends of the population will inevitably destroy the required precision (Fig 1A illustrates this schematically). Chemical synapses generally show low- or band-pass transfer functions. Comparing the impact of temporal low-pass filtering at chemical synapses (Fig 9, *σ*_*kernel*_) and that of (conduction) delays (*σ*_*delay*_) one can observe that mutual information measures are higher above the identity line and the drop-off is steeper for *σ*_*delay*_ than for *σ*_*kernel*_. The spread of conduction delays therefore appears to be more detrimental than the low-pass filtering at a chemical synapse.

### Integration in the ELL balances delays and information gain

In *A. leptorhynchus* P-units project to the electrosensory lateral line lobe (ELL) [30]. There, the target pyramidal cells in the three adjacent maps of the ELL [28, 35, 39] have different sign preferences, receptive field sizes, and spectral tuning properties [38, 39, 69]. Receptive fields are largest in the lateral segment where pyramidal cells integrate about 1000 P-units [39]. Peripheral information is relayed to ELL pyramidal cells via relatively fast chemical synapses. From *in vitro* studies on these we can estimate the time-constants of the low-pass component of the EPSPs [70]. The horizontal and vertical dashed lines in Fig 9 depict lower and upper estimates of the receptive field sizes and synaptic kernels in the lateral segment. The intersection area highlights the actual combination. It appears that especially the receptive field size is a good compromise for maximizing the gain from population coding and minimizing the loss due to conduction delays. The current view on the ELL is that lateral segment cells encode high-frequency global signals that arise from social interactions while, on the other extreme, the cells in the centro-medial segment with small receptive fields (about 10 P-units), and higher sensitivity for low-frequency signals, are devoted to the processing of navigation and prey-related signals. Confusingly, small receptive fields would be particularly well suited to encode high frequency signals as attenuation by conduction delay spread is minimal. It was previously found that P-units not only encode the AM in their firing rate but that spikes simultaneously carry precise timing information [50]. Timing information was found to be conserved in pyramidal neurons in the lateral segment, though to a lesser extent than in the input layer. Future studies should address whether smaller receptive fields in the centro-medial segment conserve timing information better than the larger receptive fields in the lateral segment.

### Possible compensatory mechanisms

Here we modelled the integration of electrosensory information as an average across the presynaptic activity (see also [5]). This is the simplest readout mechanism but sufficient for the point we want to make. It is known, however, that electrosensory information undergoes a transformation when relayed to the ELL [30] and the nervous system could in principle compensate for such delays in several ways. First, by selectively increasing the conduction velocity for distant fibers. But, as mentioned above, the available data does not support the existence of such a mechanism. Second, similar adaptations could be done on the input dendrites of the pyramidal neurons, but to the best of our knowledge, there is no evidence for such adaptations. Third, delayed inputs could be suppressed by fast inhibitory currents, mediated by SK-potassium channels [71] and found to be critical for the high-pass tuning in LS pyramidal cells [72]. Another option could be a selective readout of simultaneous afferents. In the torus semicircularis, which receives electrosensory input from the ELL, recent findings hint at a weighted readout mechanism [73] favoring certain presynaptic inputs over others. Work from the auditory cortex shows that population information is maximized when favoring neurons that fire sparsely, but precisely, and thus implement a weighted integration [74]. On the processing level considered here, however, we have no evidence for such a selective readout mechanism and the stimulus encoding in P-units is dense, instead of sparse.

## Conclusion

In their great book on the principles of neural design Sterling and Laughlin work out three main principles that define the way nervous systems should be designed [75]: (i) Only the absolutely required information should be sent down axons, (ii) sending information should employ as few action potentials as possible and (iii) the system should be designed to minimize the use of axons in terms of length and diameter. Here we would like to add considerations about the compromise of encodable stimulus dynamics, receptive field size, and information (i.e. the signal to noise ratio).

Reading out information from a sensory surface must be a compromise of the receptive field size (the population size) and the signal dynamics that need to be encoded. This again emphasizes the neuroethological claim that we can only understand the brain when we consider it within its natural context with knowledge about the natural stimuli and the extracted features [76–79].

## Supporting information

S1 Fig Experimental setup. Schematics of the side and top view of the experimental tank. Numbers refer to the recorded signals depicted on the right. **1**: The semi-intracellular potential was recorded in the lateral line nerve. **2–4** The electric field of the fish was recorded in three different ways. From top to bottom; **2**: *Global EOD* head to tail measurement, measurement electrodes were placed isopotential to the stimulus electrodes to record the unperturbed field of the fish. **3**: The *Reference EOD* is a proxy of the transdermal potential picked up by the electroreceptor afferents and is measured using a pair of silver wired oriented orthogonal to the body axis of the fish and placed just posterior of the operculum. **4**: Local EOD measurement dipole mounted on the robot arm. Via two carbon rods (**5**) the global stimulus could be given. A local stimulus was given via the dipole electrode **6**.
(TIF)

S2 Fig Estimating receptor position. The location of a recorded P-unit on the body of the fish was estimated by moving the local stimulus dipole alongside the rostro-caudal axis of the animal. At each position a stimulus was presented that led to an amplitude modulation of the recorded fish’s EOD (bottom trace, the blue line is the carrier, i.e. the fish’s EID, the red line indicates the induced amplitude modulation with the period *p*). The neuronal spiking response (panels in row 2) were measured and the firing rate was estimated using kernel convolution with a Gaussian kernel (red line). The power at the expected frequency *p*^−1^ was extracted from the power spectra of the firing rate (panels in row 3) and was then plotted as a function of the rostro-caudal position (panel 4). The receptor location was the maximum position of a fitted Gaussian.
(TIF)

S3 Fig Correlations of cellular response properties. Color codes for the Pearson correlation coefficient ‘r’ with blue colors depicting positive and red colors depicting negative correlations. p-values are Bonferroni corrected (n = 15 correlations). A statistically significant correlation was assumed for p < 0.05.
(TIF)

S4 Fig Stimulus encoding with homogeneous and heterogeneous populations. **A**: Responses of four example cells to the same white noise stimulus. The cells were chosen based on similar the average firing rates (100 to 15 Hz) and similar response modulations (40 to 60 Hz). Left: The raster plot in the background depict the spike times of up to 10 consecutively recorded stimulus repetitions (150 ms out of 10 s total trial duration). Orange trials were randomly selected to create the population response shown in B. Blue line depicts the across-trial firing rate estimated by kernel convolution with a Gaussian kernel with a standard deviation of 1.25 ms. Right: stimulus response coherence smoothed with a five point running average and based on segments of 16384 samples (0.82 s) and 50% overlap. Mutual information is calculated according to Eq 8. **B**: Population response of a “homogeneous” population created as the average of the orange-highlighted trials in A. **C**: Same as A, but for four example cells with different response properties. Maximum and minimum firing rate (top and second row) minimum and maximum response modulation (third and fourth row). **D**: Same as B but trials were selected from the heterogeneous cells shown in C.
(TIF)
