# Supplementary figures and images for: Receptive field sizes and neuronal encoding bandwidth are constrained by axonal conduction delays
Source: PLoS Comput Biol. 2023 Aug 11;19(8):e1010871. doi: 10.1371/journal.pcbi.1010871 (PMC10446211; doi:10.1371/journal.pcbi.1010871)

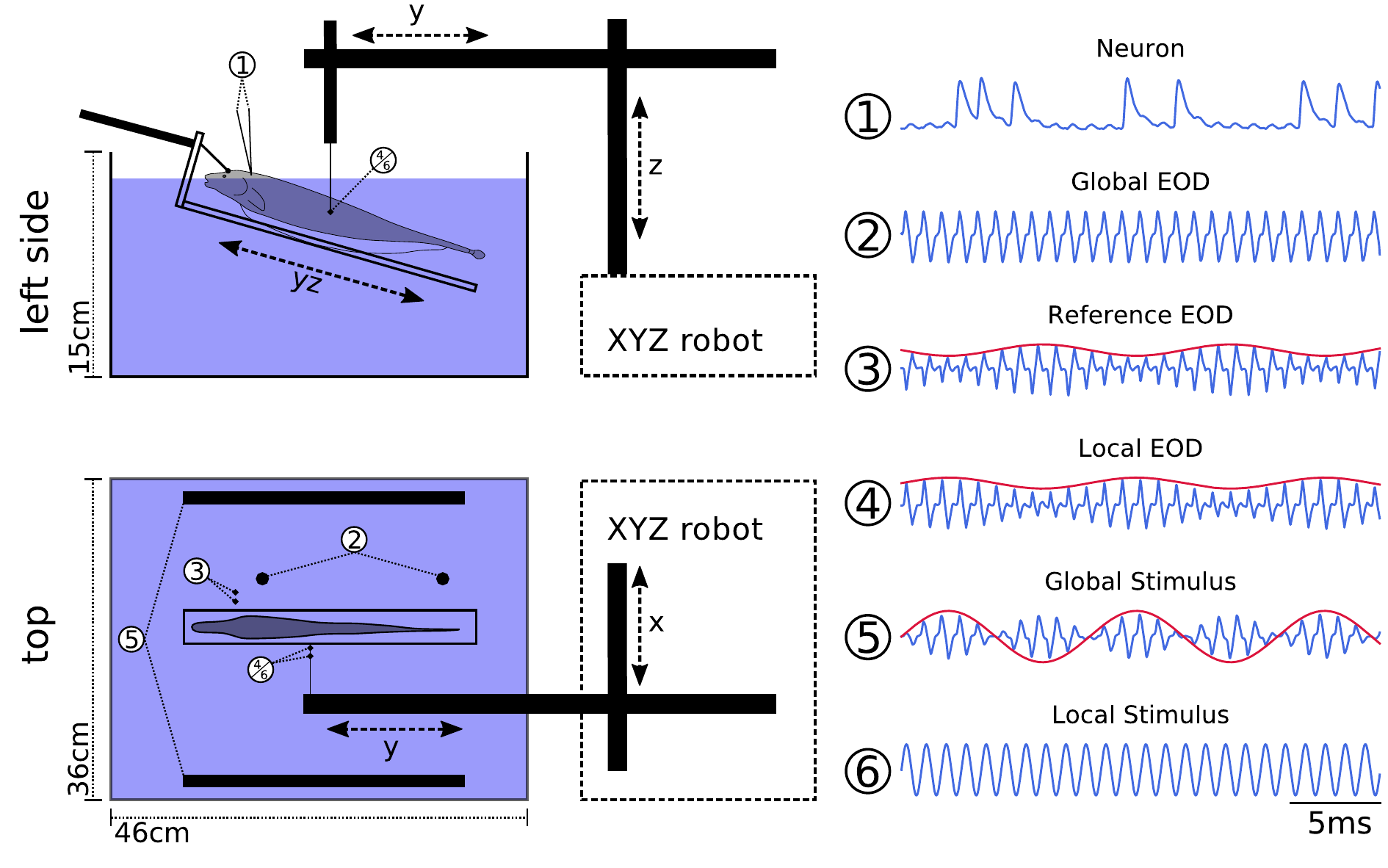

Supplement: S1 Fig — Schematics of the side and top view of the experimental tank. Numbers refer to the recorded signals depicted on the right. 1: The semi-intracellular potential was recorded in the lateral line nerve. 2–4 The electric field of the fish was recorded in three different ways. From top to bottom; 2: Global EOD head to tail measurement, measurement electrodes were placed isopotential to the stimulus electrodes to record the unperturbed field of the fish. 3: The Reference EOD is a proxy of the transdermal potential picked up by the electroreceptor afferents and is measured using a pair of silver wired oriented orthogonal to the body axis of the fish and placed just posterior of the operculum. 4: Local EOD measurement dipole mounted on the robot arm. Via two carbon rods (5) the global stimulus could be given. A local stimulus was given via the dipole electrode 6. (TIF) [file pcbi.1010871.s001.tif]

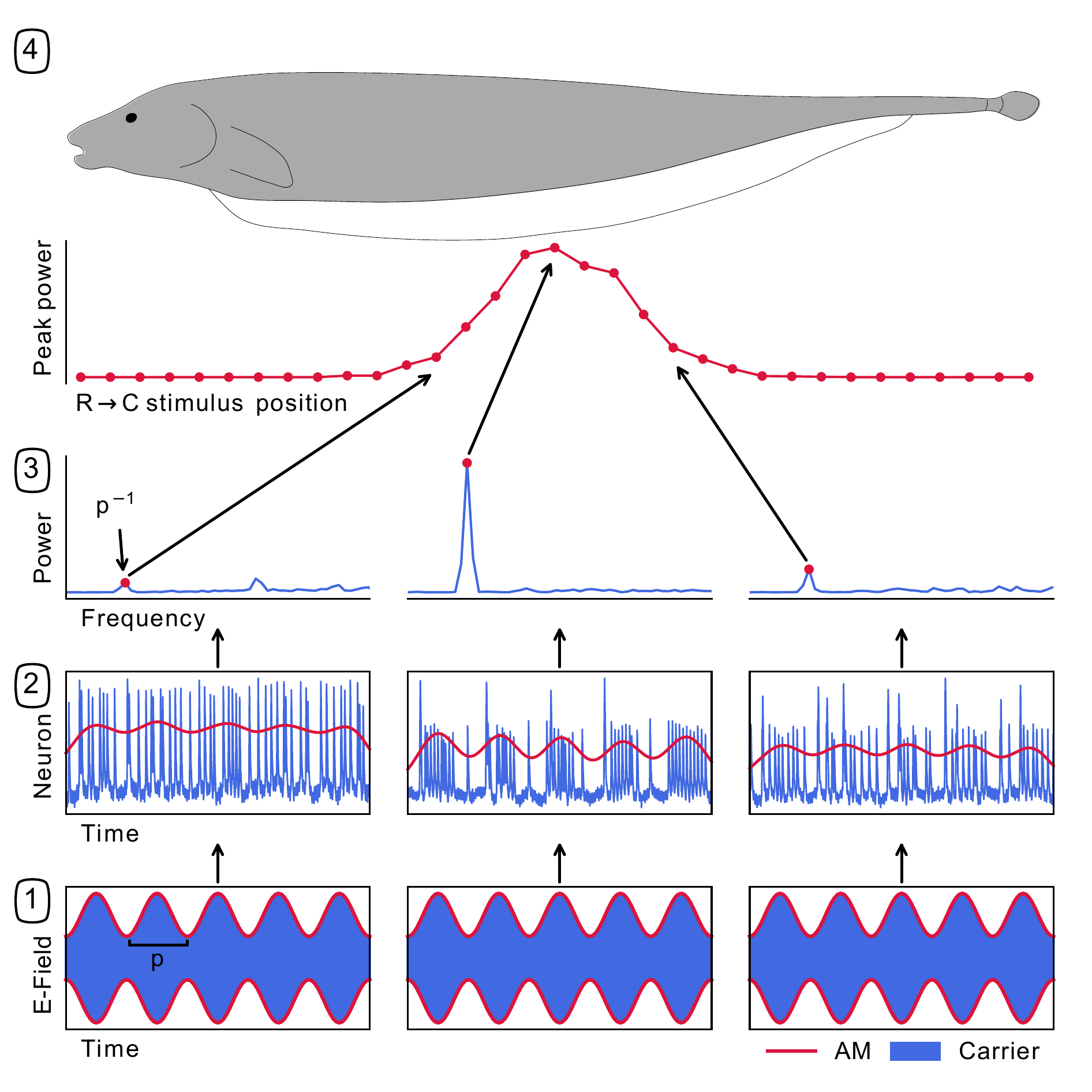

Supplement: S2 Fig — The location of a recorded P-unit on the body of the fish was estimated by moving the local stimulus dipole alongside the rostro-caudal axis of the animal. At each position a stimulus was presented that led to an amplitude modulation of the recorded fish’s EOD (bottom trace, the blue line is the carrier, i.e. the fish’s EID, the red line indicates the induced amplitude modulation with the period p). The neuronal spiking response (panels in row 2) were measured and the firing rate was estimated using kernel convolution with a Gaussian kernel (red line). The power at the expected frequency p−1 was extracted from the power spectra of the firing rate (panels in row 3) and was then plotted as a function of the rostro-caudal position (panel 4). The receptor location was the maximum position of a fitted Gaussian. (TIF) [file pcbi.1010871.s002.tif]

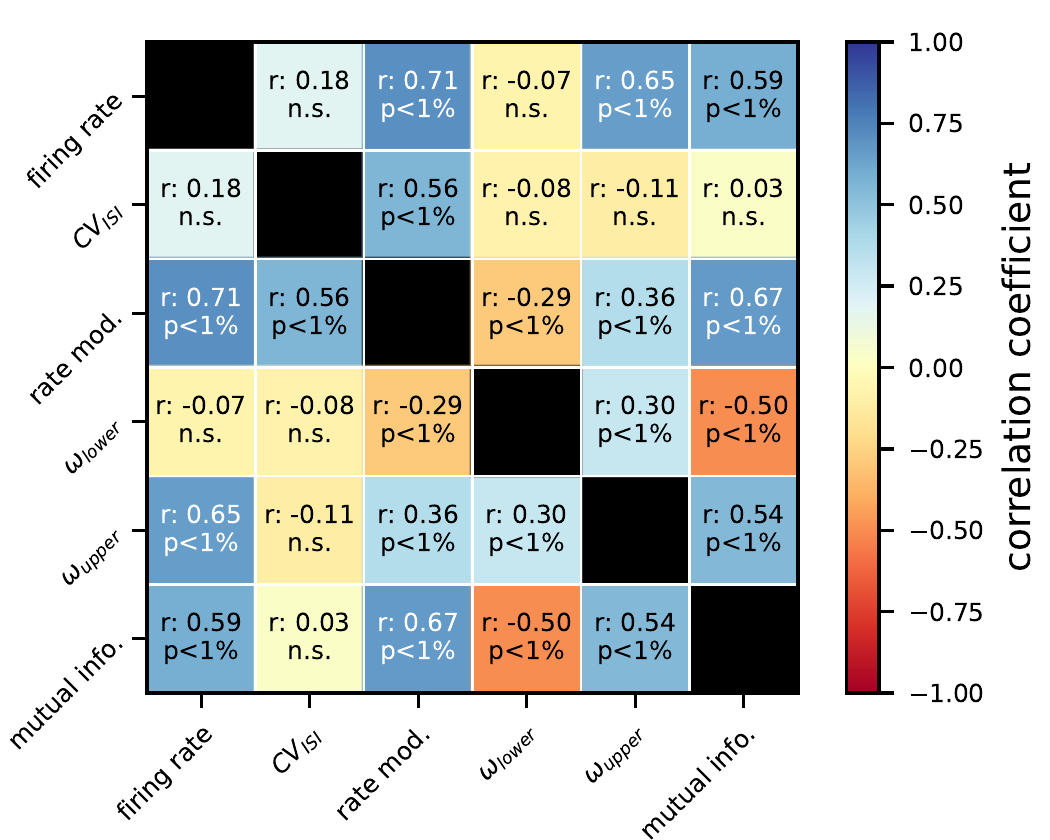

Supplement: S3 Fig — Color codes for the Pearson correlation coefficient ‘r’ with blue colors depicting positive and red colors depicting negative correlations. p-values are Bonferroni corrected (n = 15 correlations). A statistically significant correlation was assumed for p < 0.05. (TIF) [file pcbi.1010871.s003.tif]

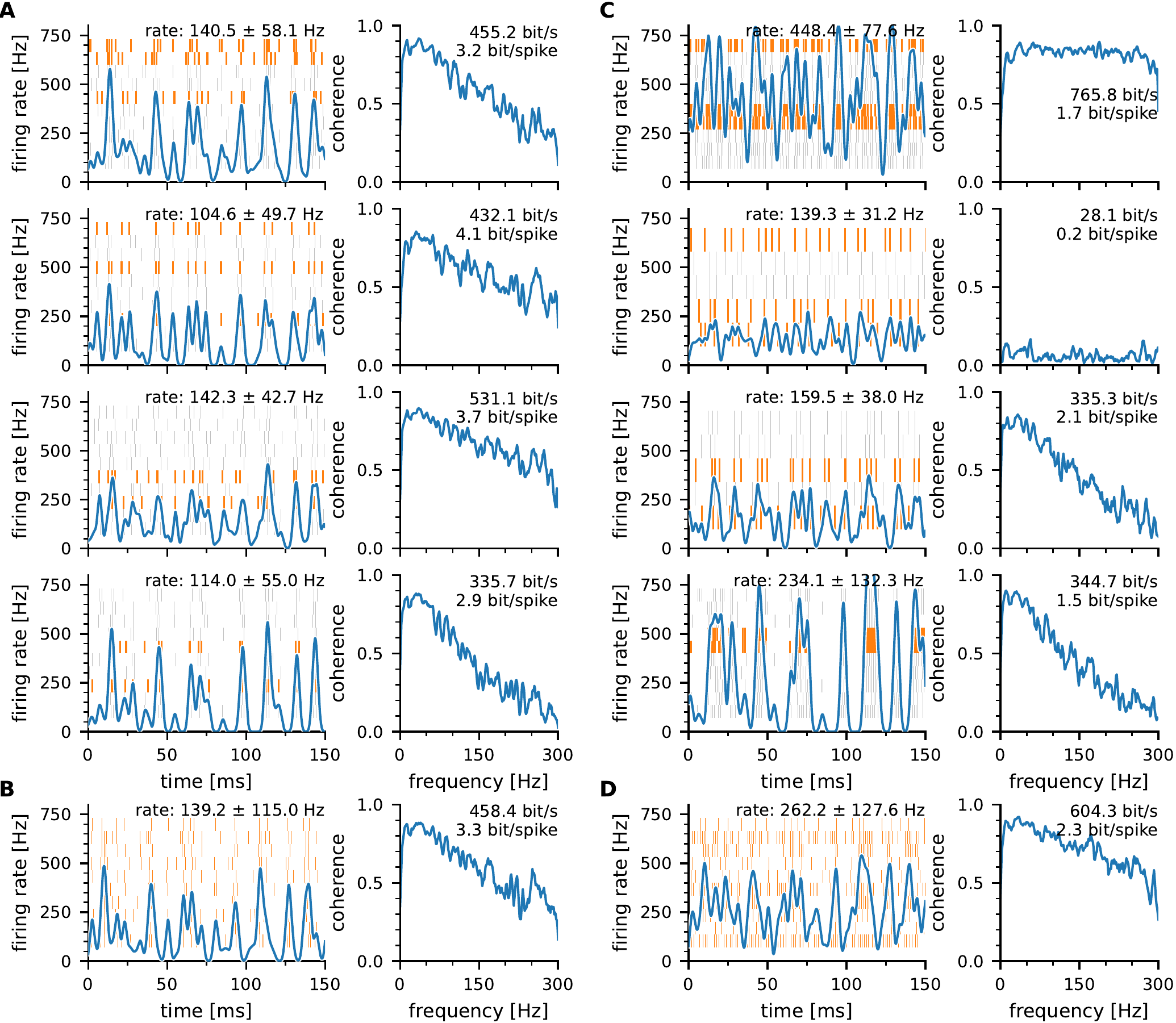

Supplement: S4 Fig — A: Responses of four example cells to the same white noise stimulus. The cells were chosen based on similar the average firing rates (100 to 15 Hz) and similar response modulations (40 to 60 Hz). Left: The raster plot in the background depict the spike times of up to 10 consecutively recorded stimulus repetitions (150 ms out of 10 s total trial duration). Orange trials were randomly selected to create the population response shown in B. Blue line depicts the across-trial firing rate estimated by kernel convolution with a Gaussian kernel with a standard deviation of 1.25 ms. Right: stimulus response coherence smoothed with a five point running average and based on segments of 16384 samples (0.82 s) and 50% overlap. Mutual information is calculated according to Eq 8. B: Population response of a “homogeneous” population created as the average of the orange-highlighted trials in A. C: Same as A, but for four example cells with different response properties. Maximum and minimum firing rate (top and second row) minimum and maximum response modulation (third and fourth row). D: Same as B but trials were selected from the heterogeneous cells shown in C. (TIF) [file pcbi.1010871.s004.tif]
